# Deciphering the role of female reproductive tract microbiome in reproductive health: a review

**DOI:** 10.3389/fcimb.2024.1351540

**Published:** 2024-03-18

**Authors:** Hong Gao, Qiao Liu, Xiaolan Wang, Ting Li, Huanhuan Li, Genlin Li, Lingling Tan, Yahui Chen

**Affiliations:** ^1^ Nursing Department, The Second Affiliated Hospital, Hengyang Medical School, University of South China, Hengyang, China; ^2^ Ottawa Hospital Research Institute, The Ottawa Hospital, Ottawa, ON, Canada; ^3^ School of Nursing, University of South China, Hengyang, China; ^4^ Center for a Combination of Obstetrics and Gynecology and Reproductive Medicine, The First Affiliated Hospital, Hengyang Medical School, University of South China, Hengyang, China; ^5^ Department of Obstetrics, The Second Affiliated Hospital, Hengyang Medical School, University of South China, Hengyang, China; ^6^ Department of Gynaecology, The Second Affiliated Hospital, Hengyang Medical School, University of South China, Hengyang, China

**Keywords:** microbiome, reproductive health, female reproductive tract, anatomy, histology, immunity

## Abstract

Relevant studies increasingly indicate that female reproductive health is confronted with substantial challenges. Emerging research has revealed that the microbiome interacts with the anatomy, histology, and immunity of the female reproductive tract, which are the cornerstone of maintaining female reproductive health and preventing adverse pregnancy outcomes. Currently, the precise mechanisms underlying their interaction and impact on physiological functions of the reproductive tract remain elusive, constituting a prominent area of investigation within the field of female reproductive tract microecology. From this new perspective, we explore the mechanisms of interactions between the microbiome and the anatomy, histology, and immunity of the female reproductive tract, factors that affect the composition of the microbiome in the female reproductive tract, as well as personalized medicine approaches in managing female reproductive tract health based on the microbiome. This study highlights the pivotal role of the female reproductive tract microbiome in maintaining reproductive health and influencing the occurrence of reproductive tract diseases. These findings support the exploration of innovative approaches for the prevention, monitoring and treatment of female reproductive tract diseases based on the microbiome.

## 1 Introduction

The female reproductive tract is an important microecological region, similar to other mucosal sites, where a wide variety of microbial communities colonize and proliferate. These communities antagonize, promote, and coexist with the female reproductive tract mucosa, forming a complex reproductive tract microecosystem (Chen et al., 2017). In the course of researching the female reproductive tract microecology, it was found that the microbiome, as a vital member of the reproductive tract microecology, interacted with the anatomy, histology, and immunity and had great potential in maintaining reproductive health (Muzny et al., 2020; Wira et al., 2005; Zhu et al., 2022).

In recent years, with the appearance and application of next-generation sequencing (NGS) technology, research on the composition of the female reproductive tract microbiome has been rising rapidly (Koedooder et al., 2019). The unique and important microbial communities in the different parts of the female reproductive tract have gradually been confirmed, but no consensus exists on their composition (Venneri et al., 2022). It is essential to understand whether they are resident or pathogenic bacteria, as they have a crucial impact on the health and diseases of the female reproductive tract (Venneri et al., 2022; Łaniewski et al., 2020). In a balanced female reproductive tract microecology, the mucosa, optimal pH, and appropriate immune response provide favorable conditions for the colonization of the tissue-resident microbiome (Wira et al., 2005; Anderson et al., 2014). Cervicovaginal *Lactobacillus* strengthen the epithelial barrier to prevent the invasion of pathogenic bacteria, thereby reducing reproductive tract infections and maintaining reproductive tract health (Delgado-Diaz et al., 2022; Anton et al., 2017; Anton et al., 2018). Endometrial tissue-resident microbiome, such as *Lactobacillus* and *Bacteroides*, compete with pathogenic bacteria for ecological niches and may regulate maternal-fetal immune tolerance, which is conducive to protecting the upper reproductive tract from pathogenic bacteria and embryo implantation (Moreno et al., 2016; Kim et al., 2019; Mazmanian et al., 2005). The mucosa of the reproductive tract can also sense pathogenic bacteria to promote the growth, maturation, and differentiation of immune cells and be engulfed by immune cells; subsequently, immunoactive cells produce immune factors and split target cells to effectively eliminate pathogenic bacteria and maintain epithelial barrier integrity (Wira et al., 2005; Yarbrough et al., 2015). When a large number of pathogenic bacteria accumulate in the female reproductive tract, it can disrupt maternal-fetal immune tolerance and induce premature cervical remodeling, ultimately endangering embryo implantation, fetal development and delivery (Inversetti et al., 2023; Elovitz et al., 2019). As a consequence, the female reproductive tract harbors a diverse microbial community that exerts a significant influence on its physiological function. These interactions between the microbiota and the anatomy, histology, and immunity maintain a dynamic balance in the microenvironment of the female reproductive tract and affect its health and disease.

The composition of the female reproductive tract microbiota is influenced by various host and environmental factors, which are common and easily coexist (Łaniewski et al., 2020; Wang et al., 2022). In the interaction with the host and the environment, changes in the microbiota may shape physiological or pathological alterations in the internal environment of the reproductive tract (Łaniewski et al., 2020). The increasing age of women, changes in menstrual cycles, and fluctuations in estrogen levels often cause physiological alterations in the reproductive tract microbiota (Gajer et al., 2012; Wang et al., 2021). Long-term exposure to adverse host and environmental factors can lead to dysregulation of the microbiota and its pathological changes in the reproductive tract, which may ultimately cause female reproductive tract diseases (Łaniewski et al., 2020; Ruff et al., 2020). Researchers have found that reducing microbial imbalance and increasing the abundance of beneficial bacteria can improve reproductive health and treat female reproductive tract diseases (Recine et al., 2016; Cohen et al., 2020; Huang, 2017; Iwami et al., 2023). Indeed, modulating the microbial composition to restore microecological balance is expected to become a new pathway for managing female reproductive tract health.

These findings suggest that microbial communities are a crucial presence in the health and disease of the female reproductive tract, and they deserve a systematic review. Therefore, we first reviewed the microbiota composition in each part of the female reproductive tract and the latest developments in microbiome detection technologies. Second, by reviewing a large number of studies, the potential mechanisms of the interactions between the microbiota and the anatomy, histology, and immunity of the female reproductive tract were explored, with a specific focus on elucidating the impact of this interaction on the physiological functions of the reproductive tract. A comprehensive overview of the factors that affected the female reproductive tract microbiome composition was provided afterwards. Finally, we discussed personalized medicine approaches in managing female reproductive tract health based on microbiome.

## 2 Composition of the female reproductive tract microbiome

**Figure 1:**
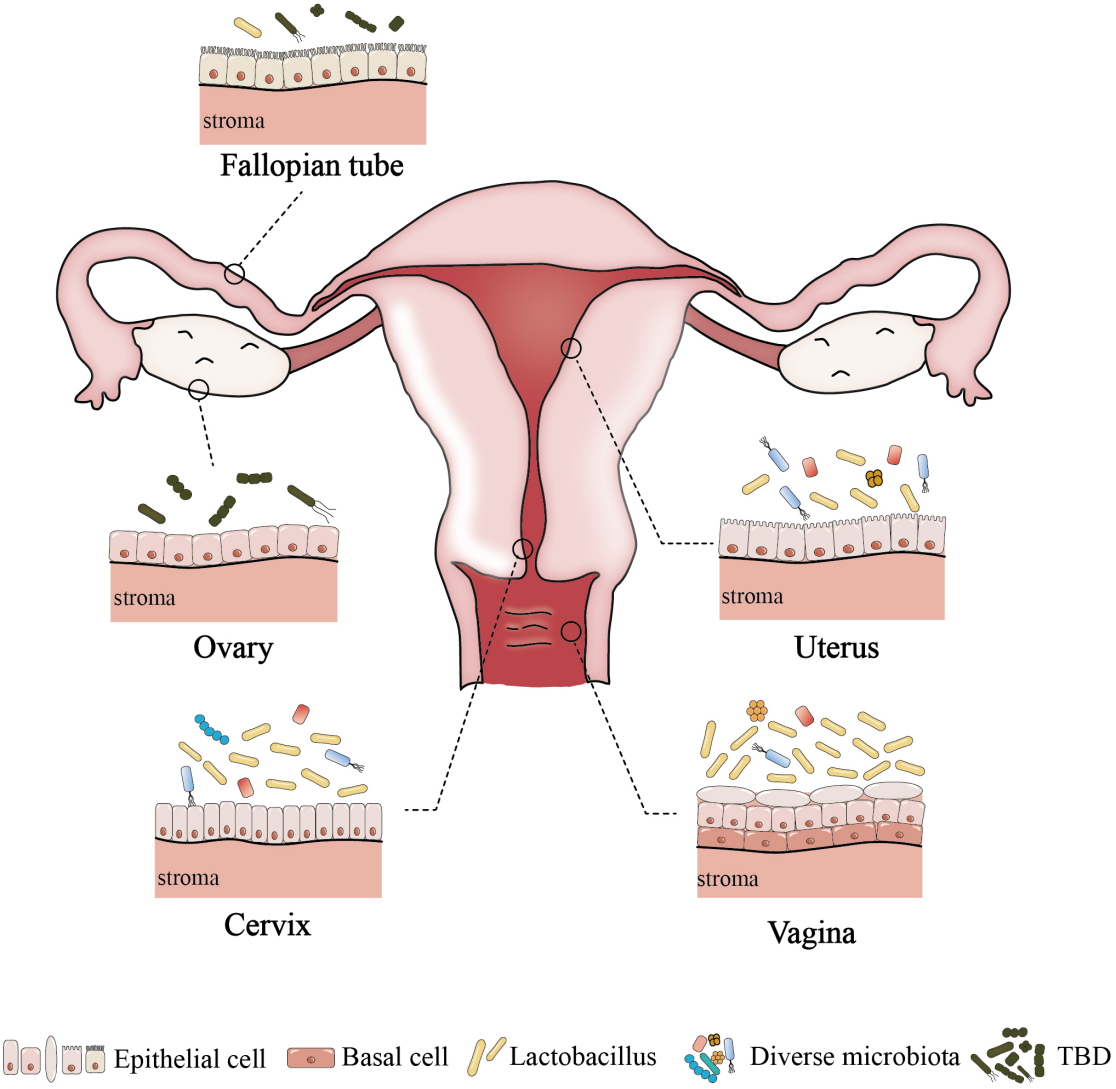
The difference of microenvironment in different parts of female reproductive tract. Among healthy women of childbearing age, the lower reproductive tract is low microbial diversity. In contrast, the uterus has a higher microbial diversity,and the abundance of *Lactobacillus* is lower. The upper reproductive tract may contain a small number of microbes, but the resident microbiome of fallopian tubes and ovaries has not been determined. TBD, to be determined.

Anatomically, the female reproductive tract is divided into the lower reproductive tract (vagina and cervix) and the upper reproductive tract (uterus, fallopian tubes and ovaries), which are connected to the external environment. Studies on the microbiome of the reproductive tract have confirmed that there is colonization by a microbial community, rather than it being a sterile area (Chen et al., 2017; Łaniewski et al., 2020). An astonishing phenomenon has been uncovered, whereby despite the interconnectivity of the reproductive tract, a discernible difference exists in the microbial communities between the respective parts; and from the lower reproductive tract to the uterus, the relative abundance of Lactobacillus and the bacterial biomass gradually decrease, while the microbial diversity progressively increases (**Figure 1**) (Chen et al., 2017; Łaniewski et al., 2020). And the specific microbiome composition in different parts of the female reproductive tract is described below.

### 2.1 Microbiome composition of the lower reproductive tract

#### 2.1.1 Vaginal microbiome

As the entrance part of the female reproductive tract, the vagina harbors the highest bacterial biomass, and it has been confirmed that there is a resident microbiome in the vagina (Chen et al., 2017; Ravel et al., 2011; Integrative, 2019). At the genus level, there is a higher relative abundance of *Lactobacillus* (more than 89%), while the presence of *Prevotella*, *Sneathia*, *Staphylococcus*, *Veillonella*, *Streptococcus* and others is still controversial (Chen et al., 2017; Ravel et al., 2011; Santella et al., 2022). Some scholars believe that the dominance of *Lactobacillus* in the vaginal microbiome represents a healthy, normal microbial environment, and the relative abundance of the dominant *Lactobacillus* determines the type of bacterial community, which is called the community state types (CSTs) (Kroon et al., 2018; Lloyd-Price et al., 2017; Younes et al., 2018). The CSTs are divided into five distinct CSTs, namely, CST I (dominated by *L. crispatus*), CST II (dominated by *L. gasseri*), and CST III (dominated by *L. iners*), CST IV [polymicrobial microbiome including *Lactobacillus* and BV-associated bacteria (BVAB)], CST V (dominated by *L. jensenii*) (Lloyd-Price et al., 2017; France et al., 2020). Currently, CSTs I, III, and IV, which have been extensively studied, are common in women, but CSTs II and V are rarely found (Doyle et al., 2018). Studies have shown that a vaginal microbiome dominated by *L. crispatus* (CST I) always maintains vaginal health, whereas a vaginal microbiome dominated by *L. iners* (CST III) is more prone to vaginal diseases (Jakobsson and Forsum, 2007; Verstraelen et al., 2009). Specific microbial taxa in the vagina can affect vaginal health and diseases by regulating inflammatory factors and their metabolites, hence necessitating an in-depth investigation of their interrelationship (De Seta et al., 2019; Breshears et al., 2015; Fuochi et al., 2019).

#### 2.1.2 Cervical microbiome

Over the years, it has been widely believed that the cervical microbiome is a continuation of the vaginal microbiome; however, recent evidence has confirmed differences between the vaginal and cervical microbiome (Chen et al., 2017). It has been found that among the cervical microbiome, *Firmicutes* is considered to be the most abundant phylum, and *Lactobacillus* is the main genus in this phylum (as high as 80.2%) (Onywera et al., 2019a; Onywera et al., 2019b). Keburiya et al. demonstrated that *L.crispatus* in the cervix could produce lactic acid and antimicrobial compounds, inhibit inflammation, thereby reducing the incidence of human papilloma virus (HPV) infection (Keburiya et al., 2022). *Bacteroidetes* is the second most abundant phylum, of which *Prevotella* is the dominant genus. *Prevotella*, as an important member of the cervical microbiome, affects the development of cervical lesions and persistent HPV infection through host nuclear factor kappa B (NF-κB)/C-myc during HPV infection in women of reproductive age (Dong et al., 2022). The next most abundant phyla are *Actinobacteria* and *Fusobacteria*, with *Gardnerella* and *Sneathia* being the most abundant genera, respectively (Onywera et al., 2019a; Onywera et al., 2019b). Intriguingly, higher abundances of *Gardnerella* and *Sneathia* were found in high-risk HPV-infected women compared to low-risk HPV-infected or non-HPV-infected women, suggesting that they may be closely related to high-risk HPV infection (Onywera et al., 2019a). It is imperative to enhance our understanding of the composition of the cervical microbiome and explore it plays a pivotal role in the screening and diagnosis of cervical HPV infection.

### 2.2 Microbiome composition of the upper reproductive tract

#### 2.2.1 Endometrial microbiome

Throughout the past century, the uterine cavity has traditionally been perceived as a sterile environment devoid of microbial colonization (Heinonen et al., 1985). However, accumulating evidence suggests a low abundance and high diversity of microbiome colonize the endometrium (Moreno et al., 2016; Chen P. et al., 2021; Franasiak et al., 2016; Verstraelen et al., 2016). Some studies showed that *Lactobacillus*, *Sphingobium*, *Acinetobacter*, *Methylobacterium* and *Streptococcus* dominated the endometrium (Chen P. et al., 2021). Other studies assumed that *Lactobacillus* and *Flavobacterium* could represent the majority of the endometrial microbiome (Franasiak et al., 2016). Moreno et al. reported that *Lactobacillus* (71.7%), *Gardnerella* (12.6%), *Bifidobacterium* (3.7%), *Streptococcus* (3.2%), and *Prevotella* (0.866%) were the most common bacteria in the endometrium (Moreno et al., 2016). There is no consensus on the core microbiome of the endometrium in healthy women, but *Lactobacillus* is a consistent discovery, and it is considered one of the endometrium-resident microbes (Moreno et al., 2016). Previous research revealed that the endometrial microbiome was dominated by *Lactobacillus* (LD, *Lactobacillus*>90%, other bacteria<10%), and the clinical pregnancy rate and live birth rate were higher, which might predict the reproductive success; when the relative abundance of *Lactobacillus* was low (*Lactobacillus*<90%, other bacteria>10%), identified as microbial dysbiosis, the incidences of adverse pregnancy outcomes such as recurrent spontaneous abortion, preterm birth, biochemical pregnancy, and recurrent implantation failure (RIF) were increased (Moreno et al., 2016). Currently, the endometrial microbiome is considered an effective biomarker for predicting reproductive success rate, which could provide new insights and research directions for the prevention and treatment of adverse pregnancy outcomes (Punzón-Jiménez and Labarta, 2021).

#### 2.2.2 Tubal microbiome

Compared to the lower reproductive tract and the uterus, there are relatively few studies on the tubal microbiome (Miles et al., 2017; Zhou et al., 2019; Walther-António et al., 2016; Pelzer et al., 2018a; Canha-Gouveia et al., 2023). Walther-António et al. studied the whole reproductive tract microbiome of 31 patients with total hysterectomy and bilateral adnexectomy. The results revealed that *Shigella* and *Bacteroides* were the most important taxa in the fallopian tube (Walther-António et al., 2016). A study of the tubal microbiome based on salpingectomy showed that the main taxa included *Staphylococcus*, *Enterococcus*, *Corynebacterium* and *Lactobacillus* (Pelzer et al., 2018a). On the whole, the fallopian tube contains a variety of bacteria suitable for growth in a weakly alkaline environment, and the proportion of *Lactobacillus* is extremely low, which is markedly different from the microbiological composition of the lower reproductive tract and endometrium (Chen et al., 2017; Punzón-Jiménez and Labarta, 2021; Peric et al., 2019). In most studies on the tubal microbiome, the selected subjects tended to have benign diseases of the uterus and cervix that may affect the cervical physiological barrier, making it easy for bacteria (such as *Lactobacillus*) that colonize the lower reproductive tract to migrate upward (Walther-António et al., 2016; Pelzer et al., 2018a). A recent study comparing the microbiome of the fallopian tube in women who underwent tubal ligation (normal controls) and those who underwent hysterectomy for benign disease (cases) showed no significant differences in microbial diversity or differential abundance analysis (Canha-Gouveia et al., 2023). It was also found that the most prevalent genera among fallopian tube samples were *Lactobacillus*, *Prevotella*, *Acinetobacter*, *Propionibacterium*, and *Faecalibacterium* (Canha-Gouveia et al., 2023). This study is a significant impetus for further investigation into the microbial communities within the fallopian tubes. In the future, the normal composition of the tubal microbiome should be further explored to enrich the study of upper genital tract microbiome composition and reach a consensus on the composition of the tubal microbiome as soon as possible.

#### 2.2.3 Ovarian microbiome

Until now, studies on the ovarian microbiome have hardly been described, mainly focusing on patients with gynecological tumors, and the ovarian microbiome of normal individuals has been particularly rare (Miles et al., 2017; Zhou et al., 2019; Banerjee et al., 2017). In a recent study, *Corynebacterium*, *Blautia*, *Escherichia*, *Lactobacillus*, and *Trabulsiella* were found to be highly enriched, while *Lactobacillus* was significantly decreased in ovarian samples from patients with malignant disease (Miles et al., 2017). Compared to normal ovarian samples, ovarian cancer samples exhibited significant differences in the composition of their ovarian microbiome. *Pediococcus* was the most commonly detected microbe, followed by *Acinetobacter*, *Staphylococcus*, *Sphingomonas*, *Enterococcus*, *Chryseobacterium*, and *Burkholderia* (Banerjee et al., 2017). Furthermore, the microbiome in malignant ovarian tissue displayed distinct microbial signatures when compared to the healthy surrounding ovarian tissues within the same individuals. Specifically, potentially pathogenic intracellular microorganisms, such as *Acinetobacter*, *Chlamydia* and *Mycoplasma*, were detected in 60%~76% of ovarian cancer cases (Zhou et al., 2019; Banerjee et al., 2017). These studies indicated that the reduction or disappearance of *Lactobacillus* in the ovarian microbiome and the increase in certain bacteria in the ovarian microbiome could potentially serve as biosignatures for the presence of gynecological tumors. Similarly, normal ovarian tissue has been found to be colonized by microbes (Zhou et al., 2019; Banerjee et al., 2017). So, what are the normal microbial communities found in the ovaries? The answer remains elusive due to the challenges in obtaining normal ovarian samples.

### 2.3 Diagnostics advancements in technologies for detecting the microbiome

Historically, the cultivation of the microbiome requires specific conditions, including biochemically defined media, precise incubation temperature, an anaerobic environment, and optimal pH levels; not all bacteria can be detected using conventional cultivation methods (Vartoukian et al., 2010; Wade, 2002). Furthermore, the cultivation of the microbiome is susceptible to various cultivation conditions, human operation, and environmental factors, resulting in a relatively high failure rate (Mashyn et al., 2022). With the appearance and application of diverse microbiome detection technologies, more and more types of microorganisms can be detected, providing information at the species and even strain level, with a trend towards quantification. These detection techniques are more accurate, easier to operate, and less affected by external interference (Gardner et al., 2010; Chiu and Miller, 2019; Khachatryan et al., 2020; Akaçin et al., 2022). We have comprehensively summarized the latest developments in microbiome detection technologies in terms of their principles, applications, advantages, and shortcomings, as presented in **Table 1**.

**Table 1:** Comparison of microbiome detection techniques.

Methods	Principles	Applications	Advantages	Shortcomings	References
Cultivation	Microorganisms can grow under certain conditions	Identification of active microorganisms	Economic, effective	Time-consuming and laborious; not all bacteria can be cultivated	(Vartoukian et al., 2010; Wade, 2002)
PCR	Polymerase chain reaction in biology	Applied to diagnostic microbiology	High sensitivity, easy to use, short turnaround time	This cannot be used to distinguish between dead and living organisms; not suitable for identifying novel microbes	(Deng et al., 2012; El-Kafrawy et al., 2021)
DNA microarrays	Labeled DNA fragments are hybridized with large-scale complementary probes fixed on slides, microspheres, or beads	Pathogens identification	Simultaneously detecting bacteria, viruses, fungi, and protozoa	Only targeting known microorganisms; sensitivity lower than PCR	(Gardner et al., 2010; Wang et al., 2002; Huang et al., 2013)
FCM	Real-time quantitative flow cytometry data	Possible use for microbial community diagnosis	Rapid cell quantification, record appropriate cell biomarkers	The sample must be a single-cell suspension	(Özel Duygan and van der Meer, 2022)
16 s rRNA gene sequencing	Relies on the 16S rRNA gene as the target sequence	Widely used in revealing microbial diversity and/or phylogenetic analysis	For bacterial identification and classification analysis	Limited to bacteria and archaea, ignoring viruses and fungi	(Khachatryan et al., 2020; Janda and Abbott, 2007; Woo et al., 2008)
Next-generation sequencing (NGS)	Solid-phase bridge amplification or emulsion polymerase chain reaction, followed by microbial sequencing during synthesis	Extensively used to study microbiota	Microbial sequencing has high throughput and deep depth	Deficiencies in reading length and accuracy	(Akaçin et al., 2022; Jo et al., 2016; Boers et al., 2019; Kozińska et al., 2019)
Metagenomic next-generation sequencing (mNGS)	Deep sequencing of the complete set of nucleic acids in a given sample	Assess which organisms are present in the sample and their proportion	Includes DNA and RNA derived from bacteria, viruses, fungi and parasites; Discovery of new microorganisms	Potential cross-contamination; Clinical interpretation of mNGS reports, distinguishing carrier status/colonization and infection remain challenging; Lack of standardization	(Chiu and Miller, 2019; Yi et al., 2024)
Whole-genome sequencing (WGS)	Amplification of randomly cut DNA segments and sequencing of the entire genome	Characterization of the complete genome	Whole genome analysis; Provide information at the species and even strain level	Expensive and time-consuming; High complexity technology	(Khachatryan et al., 2020; Kozińska et al., 2019)
Third-generation sequencing (TGS)	①Nanopore electrical signal sequencing: Single-molecule nanopore DNA sequencing from Oxford Nanopore Technologies (ONT); ②Single molecule fluorescence signal sequencing:including single molecule realtime sequencing (SMRT) technology of Pacific Biosciences (PacBio)and true single molecular sequencing (tSMS) technology of Helicos Biosciences	Single-molecule sequencing is used to decipher complex microbial ecosystems	Long read long sequencing, higher nucleotide sequence resolution: no PCR amplification; It can directly identify natural base modifications and sequence the viral RNA genome in its natural state	Higher error rate than NGS	(Akaçin et al., 2022; Yi et al., 2024)

## 3 Anatomy, histology, immunity, and microbiome of the female reproductive tract and their interactions

**Figure 2:**
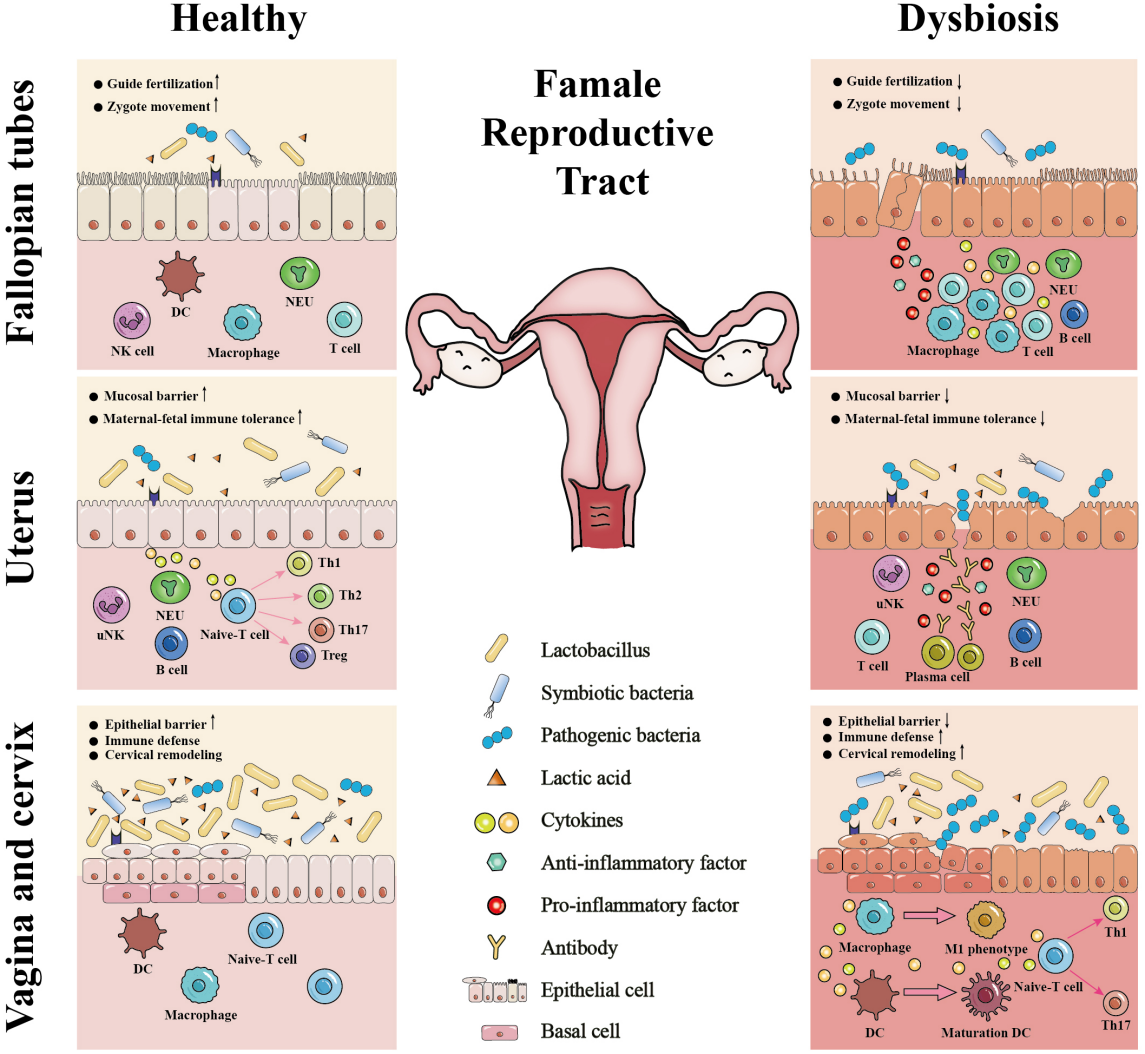
The interactions between the microbiome and the anatomy, histology, and immunity of the female reproductive tract. The *Lactobacillus*-dominant microbiome and its associated metabolites, particularly lactic acid, establish a healthy microenvironment in the female reproductive tract. This microenvironment plays a vital role in strengthening the integrity of the epithelial or mucosal barrier, stabilizing immune defense, balancing cervical remodeling, facilitating the establishment of maternal-fetal immune tolerance, guiding fertilization, and promoting the zygote movement. In contrast, microbiome dysbiosis and pathogenic bacteria invasion can 1) damage epithelial or mucosal barrier of the female reproductive tract; 2) stimulate the immune defense; 3) facilitate premature cervical remodeling; 4) disrupt maternal-fetal immune tolerance; 5) lead to tubal cilia edema, necrosis, functional decline or loss. Thus, the microbiome interacts with the anatomy, histology, and immunity of the female reproductive tract to regulate its physiological functions, including fertilization, embryo implantation, fetal development, fetal delivery and defense against pathogen infection. DC, dendritic cell; NK, natural killer; uNK, uterine natural killer; NEU, neutrophil; ↓, disrupt or damage; ↑, stimulate or facilitate.

The female reproductive tract is a continuous channel consisting of the vagina, cervix, uterus, fallopian tubes and ovaries, which is mainly involved in the birth of new life and resistance to the invasion of pathogens (Chumduri and Turco, 2021). The interactions between the microbiome and the anatomy, histology, and immunity of the female reproductive tract (except for the ovaries, as the relationships between the anatomy, histology and immunity of the ovaries and the microbiome among healthy women are also unclear) are crucial for its physiological functions (**Figure 2**). Notably, the underlying mechanisms of the effects of these interactions on physiological functions need to be further clarified, which is the cornerstone of maintaining female reproductive health and avoiding adverse pregnancy outcomes. The following sections describe their interactions in different parts of the female reproductive tract.

### 3.1 Anatomy, histology, immunity, and microbiome of the vagina and their interactions

#### 3.1.1 Anatomy, histology, and immunity of the vagina

The vagina is a muscular canal connecting the cervix to the external genitalia, with the anterior part of the vagina located near the bladder neck and urethra and the posterior part adjacent to the rectum and anus (O’Connell et al., 2008). The structure of the vagina can be divided into the vaginal epithelium (predominantly composed of multilayered stratified squamous epithelial cells), lamina propria, and fibromuscular layer from inside to outside (Mazloomdoost et al., 2017). The uppermost layer of the vaginal epithelium consists of flat cells that lack classical cell-cell adhesion, and this layer of cells undergoes rapid shedding and regeneration throughout the menstrual cycle (Anderson et al., 2014; Blaskewicz et al., 2011; Gorodeski et al., 2005; Patton et al., 2000). Furthermore, this layer of cells can use intracellular glycogen deposition to produce lactic acid through anaerobic metabolism under hypoxic conditions and secrete hydrogen ions and glycogen to the vaginal lumen through V-H^+^-ATPase (Gorodeski et al., 2005).

Vaginal mucosal immunity includes innate immunity and adaptive immunity, constituting a comprehensive defense mechanism (Bojang et al., 2021). Innate immune cells, such as vaginal epithelial cells (VECs), neutrophils (NEUs), dendritic cells (DCs), macrophages (Mφs), natural killer (NK) cells, and mast cells (MCs), express pattern recognition receptors (PRRs) on their surfaces, including Toll-like receptors (TLRs), C-type lectin receptors (CLRs), Nod-like receptors (NLRs), and RIG-I-like receptors (RLRs) (Bojang et al., 2021; Kalia et al., 2019). These PRRs interact with pathogen-associated molecular patterns (PAMPs) to activate circulating monocytes, NEUs, and Mφs in tissues (Bojang et al., 2021; Kalia et al., 2019). Among them, Mφs are commonly localized in the lamina propria of vaginal tissue, exhibiting dual functionality by secreting pro-inflammatory cytokines and modulating the formation of various inflammasome complexes (NLRP1 and NLRP3) under the action of various microbial stressors (Schroder and Tschopp, 2010). The NLRP3 inflammasome can be activated by various stimuli, including bacterial, viral, and mitochondrial damage, thereby leading to the development of bacterial vaginosis (Zhao et al., 2018; Xiang et al., 2021). Pro-inflammatory cytokines and antimicrobial peptides (AMPs) secreted by these innate immune cells, such as tumor necrosis factor (TNF)-α, interleukin (IL)-1, IL-6, IL-8, lactoferrin, human defensin-5, secretory leukocyte protease inhibitor (SLPI), elastin, ductin, and human beta-defensin-1 and -2, may be concentrated within the intracellular and/or extracellular matrix (Blaskewicz et al., 2011; Patel et al., 2013; Cole and Cole, 2008). Moreover, loosely attached VECs can create a distinct microenvironment susceptible to infiltration and penetration by resident immune cells, pro-inflammatory cytokines, and AMPs (Anderson et al., 2014). VECs also release anti-inflammatory cytokines, such as IL-10 and TGF-β, which regulate the inflammatory response and prevent excessive local cell damage (Steele and Fidel, 2002). In contrast, adaptive immunity can elicit pathogen-specific defense mechanisms by processing antigens derived from the pathogen by antigen-presenting cells (APCs) and subsequent presentation to T cells, thereby inducing T-cell activation. Following antigen presentation, antibody synthesis is activated, and cytokine production ensues. At the same time, the presence of plasma cells secreting IgG and IgA in the vagina is comparatively limited compared to the lamina propria of the cervix (Wira et al., 2005; Moragues et al., 2003).

#### 3.1.2 Effects of the interactions between the microbiome and the anatomy, histology, and immunity of the vagina on its physiological functions

Various microbes with potential commensal, symbiotic or pathogenic relationships inhabit the vagina and interact with its anatomy, histology, and immunity, thereby affecting vaginal health (Maseroli and Vignozzi, 2020; Balakrishnan et al., 2022). Endogenous *Lactobacillus* can colonize the vaginal intraepithelial and epithelial cells, enabling them to obtain nutrients and energy from glycogen stored within the epithelial cells under a slightly acidic environment (Anderson et al., 2014; Gorodeski et al., 2005). *Lactobacillus*, in turn, produces lactic acid, thereby inducing further acidification of the vaginal cavity and creating an inhospitable environment for many disease-causing bacteria and viruses (Miller et al., 2016). For example, under acidic conditions, the viability of sexually transmitted pathogens such as BVAB, *Chlamydia trachomatis*, herpes simplex virus-2, and human immunodeficiency virus significantly decreases (O’Hanlon et al., 2011; Gong et al., 2014; Conti et al., 2009; Aldunate et al., 2013). Specifically, when the pH value is below 4.5, lactic acid can induce inactivation of various BVAB (O’Hanlon et al., 2011). If there is a sufficient amount of lactic acid to lower the vaginal pH to below 4, it can efficiently inhibit *Chlamydia trachomatis* infection (Gong et al., 2014). In addition, lactic acid was observed to attenuate the release of pro-inflammatory cytokines from epithelial cells (Vallor et al., 2001). In particular, lactic acid induced the release of the anti-inflammatory cytokine and IL-1 receptor antagonist *in vitro* and inhibited the TLR-mediated production of pro-inflammatory cytokines (Chopra et al., 2022). Therefore, lactic acid reduces excessive damage to epithelial cells, which is beneficial for maintaining the integrity of vaginal epithelium. *L. crispatus* can produce both L- and D-lactic acid, leading to an increase in the levels of D-lactic acid and the ratio of D- to L-lactic acid, which has an impact on preventing upper reproductive tract infections (Witkin et al., 2013). Moreover, its production of hydrogen peroxide (H_2_O_2_) can prevent colonization by anaerobes, and there is an iron transport system in the nuclear genome of *L. crispatus*, which may hinder vaginal pathogens from obtaining iron (France et al., 2016). The microbial community dominated by *L. iners* is unstable and can only produce L-lactic acid, which is prone to transition to CST-IV (France et al., 2020). These findings suggest that *L. crispatus* may be more effective in protecting hosts from pathogens than *L. iners* (France et al., 2020; Witkin et al., 2013; France et al., 2016).

Pathogenic bacteria are also constituents of the vaginal microbiome residing in the mucosal layer. Generally, the periodic shedding of vaginal epithelial cells facilitates the elimination of pathogenic bacteria adhering to them, thereby serving as a natural defense mechanism (Patel et al., 2013; Maseroli and Vignozzi, 2020; Godha et al., 2018). In turn, pathogenic bacteria can breach the vaginal epithelial barrier. For instance, BVAB such as *Gardnerella* and *Prevotella* can produce sialidase, which potentially contributes to mucin cleavage and damage to vaginal epithelial cells (Muzny et al., 2020). VECs release pro-inflammatory cytokines, such as TNF-α, IL-1β, and IL-6, which promote the migration of local immune cells to the lesion site and initiate an immune defense to eliminate pathogenic bacteria (Balakrishnan et al., 2022; Fernando et al., 2014). When a significant number of pathogenic bacteria, such as BVAB, aggregate in the vagina and induce vaginal dysbiosis, it could increase susceptibility to sexually transmitted diseases. Pathogenic bacteria that caused intense vaginal inflammation could not only lead to increased levels of IL-1β, IL-17, and IL-23, as well as high recruitment of CCR5^+^ CD4 T cells, but they might also be strongly associated with increased susceptibility to human immunodeficiency virus (Gosmann et al., 2017). Vaginal dysbiosis induced by antibiotic treatment relies on high levels of IL-33 to suppress the adaptive immune response mediated by T cells, thereby impeding antiviral immunity against herpes simplex virus-2 infection in the mucosa (Torcia, 2019). Collectively, the interactions between the vaginal microbiome and the epithelium, as well as vaginal immunity, intricately regulated its innate and adaptive immune mechanisms, thereby governing the homeostasis or dysbiosis of the vaginal microbiome.

### 3.2 Anatomy, histology, immunity, and microbiome of the cervix and their interactions

#### 3.2.1 Anatomy, histology, and immunity of the cervix

The cervix is the gateway from the vagina to the uterus and is divided into the upper and lower parts by the top of the vagina (Ludmir and Sehdev, 2000). The upper part accounts for 2/3 of the entire cervix, and its epithelium is a single layer of tall columnar epithelium. The lower part of the cervix extends into the vagina and is covered by multilayered stratified squamous epithelium with a smooth surface (Balcacer et al., 2019). The junctions between epithelial cells include tight junctions, adherence junctions, and desmosome junctions, which form an epithelial barrier with cervical epithelial cells (Blaskewicz et al., 2011). The columnar epithelium primarily comprises tight junctions, while squamous epithelium mainly comprises adherence junctions and desmosome junctions (Wira et al., 2010). The cervical stroma is located beneath the epithelial cells and is separated from them by the basement membrane. The stroma primarily consists of an extracellular matrix supplemented by fibroblasts, immune cells, elastin, proteoglycans, and hyaluronan (Tantengco et al., 2021).

Cervical epithelial tissue is mainly composed of epithelial cells and T cells, with CD8^+^ T cells slightly more abundant than CD4^+^ T cells among the latter. The distribution of these cells is highest in the squamous and columnar transformation areas of the cervix, and lowest in the endocervix (Pudney et al., 2005). The cervix is an organ where cellular immunity predominates (Pudney et al., 2005). Once pathogenic bacteria invade, APCs recognize them through PRRs, such as TLRs and NLRs, and subsequently present the antigen to T cells, thereby initiating cellular immunity (Pudney et al., 2005). T cells are stimulated to differentiate into effector T cells, some of which develop into memory T cells. Memory T cells are divided into central memory T cells (TCMs), which are stored in the extralymphoid tissues, and effector memory T cells (TEMs), which can travel between the blood and the extralymphoid tissues and perform their functions (Gebhardt et al., 2013; Sathaliyawala et al., 2013). Some memory T cells undergo differentiation into tissue-resident memory T cells (TRMs). CD8^+^ TRMs (CD69^+^, CD103^+^) constitute the major subset of CD8^+^ T cells in cervical tissues and are also defined as inflammatory mucosal T cells (Tims). When TRMs recognized homologous peptides, they released the cytokines IFN-γ, TNF-α, and IL-2, thereby upregulating adhesion molecules and chemokines and promoting the recruitment of memory T cells and B cells to tissues (Rosato et al., 2017; Schenkel et al., 2014).

#### 3.2.2 Effects of the interactions between the microbiome and the anatomy, histology, and immunity of the cervix on its physiological functions

There is a symbiotic relationship between *L. crispatus* and the cervical mucosal epithelium; *L. crispatus* can secrete lactic acid to increase the expression of cervical epithelial barrier proteins claudin1 and claudin4 to some extent, and its supernatant can also alleviate the increase in miRNA expression induced by pathogenic bacteria (Delgado-Diaz et al., 2022; Anton et al., 2017; Anton et al., 2018). Conversely, the supernatants of pathogenic bacteria, including *Mobiluncus mulieris* and *Gardnerella vaginalis*, increased the permeability of cervical cells and the expression of miR-143 and miR-145, thereby reducing the proliferation of epithelial cells and promoting the breakdown of the cervical epithelial barrier (Anton et al., 2017; Anton et al., 2018). Researchers also have discovered that *Prevotella bivia*, *Sneathia amnii*, *Fusobacterium gonidiaformans*, and *Fusobacterium nucleatum* increase the levels of 2-hydroxyglutarate, while *Eggertella* and *Mobiluncus* decrease cysteinylglycine and cysteinylglycine disulfide levels (Łaniewski and Herbst-Kralovetz, 2021; Maarsingh et al., 2022; McKenzie et al., 2021). These findings suggested the potential contribution of cervical pathogens to necrosis and apoptosis of cervical epithelial cells through oxidative stress pathways. Additionally, cervical pathogens induced elevations of inflammatory factors such as IL-1β, macrophage inflammatory protein (MIP)-3α, and IL-8, which exhibited the strongest correlation with neutrophil proteinases [matrix metalloproteinase-9 (MMP-9) and MMP-8], suggesting potential damage to epithelial integrity (Mohammadi et al., 2022). *In vitro* culture models further confirmed that IL-1β triggered the p38 and c-Jun N-terminal kinase (JNK) signaling pathways, decreasing tight junctions and impairing epithelial integrity (Kobayashi et al., 2021). Hence, the interaction between the cervical epithelium and the microbiome can affect the function of the cervical epithelial barrier. Current research on the impact of cervical microbiome on cervical immunity primarily focuses on the recruitment and differentiation of DCs, Mφs, and T cells (Anton et al., 2022; Liu et al., 2021; Zariffard et al., 2005; van Teijlingen et al., 2020; Jan et al., 2012; Jang et al., 2017). *Gardnerella vaginalis* has been shown to activate TLR2/4 and induce an immune response in cervical cells while exerting minimal impact on DCs stimulation and promoting differentiation of M2 Mφs toward the M1 phenotype (Anton et al., 2022; Liu et al., 2021; Zariffard et al., 2005). *Megasphaera elsdenii* and *Prevotella timonensis* significantly enhanced DCs maturation and promoted T-cell differentiation towards the Th1 phenotype in the cervix (van Teijlingen et al., 2020). In contrast, cervical *Lactobacillus* inhibited the pro-inflammatory response of epithelial and immune cells, promoted the differentiation of CD4^+^ T cells into immunosuppressive regulatory T cells (Tregs), and did not affect DCs maturation (Jan et al., 2012; Jang et al., 2017). In general, the interactions of the microbiome with the cervical epithelium and immunity played crucial roles in maintaining both the epithelial integrity and immune barrier functionality within the cervix.

The cervix undergoes multiple physiological changes throughout different stages of pregnancy. Extensive remodeling of the cervix is required to allow a full-term fetus to pass through the birth canal, including softening, maturation, dilation during labor, and postnatal repair (Read et al., 2007). In the first and second trimesters, the cervix remains tightly closed to maintain pregnancy by keeping the fetus securely within the uterus (Barrios De Tomasi et al., 2019). As delivery approaches in the third trimester, the cervix gradually softens and matures, transitioning from a closed state to complete dilation, which enables smooth delivery of the fetus. After delivery, the cervix quickly returns to a tightly closed state (Read et al., 2007). Meanwhile, the physiological changes of cervical remodeling are accompanied by alterations in its stroma, such as enhanced collagen solubility and relaxation of collagen matrix (Read et al., 2007; Yellon, 2019). *Gardnerella vaginalis*, *Atopobium vaginae*, *Prevotella bivia*, and *Pseudonocardia asaccharolytica* could induce or secrete MMP-1, MMP-9, and MMP-10, while also exhibiting collagen (type I and IV) degradation abilities (Tantengco et al., 2021; Łaniewski and Herbst-Kralovetz, 2021; Lithgow et al., 2022; Short et al., 2021). An animal experiment demonstrated that the colonization of *Gardnerella vaginalis* in the reproductive tract of mice led to increased dispersion of collagen fibers, indicating accelerated cervical remodeling (Sierra et al., 2018). Conversely, *L. crispatus* did not exhibit any influence on MMP expression and type I collagen degradation (Tantengco et al., 2021; Łaniewski and Herbst-Kralovetz, 2021; Lithgow et al., 2022; Short et al., 2021). In addition, *L. iners* and *L. crispatus* can modulate the upregulation or downregulation of inflammatory bacterial signals, which may be closely associated with cervical remodeling during parturition (Anderson et al., 2014; Doerflinger et al., 2014). Taken together, symbiotic bacteria and pathogenic bacteria residing in the cervix may potentially alter the structural integrity of cervical tissue structure through induction or secretion of MMPs, collagen degradation, and inflammatory bacterial signals modulation, which affects cervical remodeling during pregnancy in humans.

### 3.3 Anatomy, histology, immunity, and microbiome of the uterus and their interactions

#### 3.3.1 Anatomy, histology, and immunity of the uterus

The uterus, a symbolic organ within the female reproductive tract, has thick-walled muscles and a hollow cavity shaped like a slightly flattened, inverted pear. Anatomically, the uterus is connected to the outside environment through the vagina and is adjacent to the rectum and bladder (Zhu et al., 2022). The uterus comprises the endometrium, myometrium, and serosa, with the endometrium serving as the initial attachment site of the embryo (Yang et al., 2019). According to the physiological structure, the endometrium is divided into two layers—the upper functional and lower basal layers. During the menstrual cycle, the upper functional layer of the endometrium is shed from the lower basal layer and then regenerated, due to changes in estrogen and progesterone (Inversetti et al., 2023). The endometrium is composed of two distinct cell types: endometrial epithelial cells, characterized by their simple columnar morphology, and endometrial stromal cells. Endometrial stromal cells transform into specialized secretory decidual cells during pregnancy, and endometrial epithelial cells also promote this process by secreting various factors (Liu et al., 2022).

The endometrium distributes a large number of immune cells, primarily uterine natural killer (uNK) cells, along with a small proportion of B cells and CD8^+^ T cells, which can generate immune mediators (Zhu et al., 2022). Immune mediators in the endometrial fluid and surface act as a barrier against direct contact between pathogens and epithelial cells while exhibiting bactericidal activity against gram-negative and gram-positive bacteria (Gershon and Dekel, 2020; Jost et al., 2014; Romero et al., 2014). It is worth noting that the number of immune cells is closely related to hormone levels, and during early pregnancy, immune cells may be as high as 30%~40% of the total number of cells in the endometrium (Zhu et al., 2022). The most abundant decidual immune cells are uNK cells, which account for 70% of the total (Yang et al., 2019). There are also Tregs, NEUs, DCs, Mφs, and MCs (Yang et al., 2019). Maintaining a normal pregnancy relies on establishing and stabilizing the maternal-fetal immune tolerance of the endometrium (Yang et al., 2019). During pregnancy, Th1 cells undergo a phenotypic switch to Th2 cells, resulting in the downregulation of the expression of Th1 cytokines IL-2, IFN-γ and TNF-α; Th17 cells transform into Tregs, and the inhibitory cytokines IL-10 and TGF-β secreted by Tregs play an immunosuppressive role (Yang et al., 2019). These mechanisms enable the fetus to evade maternal rejection throughout pregnancy, and the NF-κB signaling pathway plays a central role in regulating the immune response (Yang et al., 2019).

#### 3.3.2 Effects of the interactions between the microbiome and the anatomy, histology, and immunity of the uterus on its physiological functions

The endometrium provides a suitable area for the colonization of tissue-resident microbiome and modulates the endometrial microbiome through diverse pathways, including hormonal regulation and mucosal barriers (Zhu et al., 2022; Inversetti et al., 2023). Fluctuations in steroid hormones, such as estrogen and progesterone, during the menstrual cycle, can affect the composition of the endometrial microbiome. In particular, there are significant differences in the composition of the endometrial microbiome during the proliferative and secretory phases (Zhu et al., 2022; Inversetti et al., 2023). In the secretory phase of the menstrual cycle, endometrial epithelial cells proliferate to form a layer of adenosine cells. These adenosine cells are closely linked to create a powerful anatomical barrier that prevents resident bacteria from being exposed to the uterine immune system, thereby regulating the endometrial microbiome (Habiba et al., 2021).

Similarly, the study by Sola-Leyva et al. indicated that there is a low-biomass active microbiome in the endometrium of healthy women, which produces metabolites such as prostaglandins and tryptophan that exhibit antibacterial and immunomodulatory activities, thereby affecting endometrium function (Sola-Leyva et al., 2021). Numerous studies have consistently found that a *Lactobacillus*-dominated endometrium has a higher rate of successful embryo implantation (Moreno et al., 2016; Iwami et al., 2023). *In vitro* experiments, Kim et al. demonstrated that protein-like moieties secreted by *Lactobacillus rhamnosus* GR-1 have a unique ability to inhibit the production of pro-inflammatory cytokines in human myometrial cells, suggesting that *Lactobacillus* may inhibit maternal immune response and facilitate implantation of embryos (Kim et al., 2019). In germ-free mice colonized by *Bacteroides Fragilis*, the signaling pathway with TLR2 was activated due to the secretion of polysaccharide A (PSA), thereby inducing differentiation of Th1 cells and establishing an appropriate Th1/Th2 balance (Mazmanian et al., 2005). Therefore, it can be speculated that *Bacteroides* may modulate Th1/Th2 balance and maternal-fetal immune tolerance through PSA during pregnancy (Mazmanian et al., 2005; Inversetti et al., 2023). A high bacterial biomass or the presence of certain bacteria in the endometrium, such as *Fusobacterium* and *Jonquetella*, may be related to immune overstimulation and tissue destruction (Pelzer et al., 2018b). When the endometrial microbiome is dysbiosis, pathogenic bacteria can trigger the relevant signaling pathway to induce the release of pro-inflammatory cytokines, thus promoting the imbalance in Th17/Tregs, resulting in the RIF of the embryo (Chen P. et al., 2021). Altogether, the reciprocal interactions among the endometrium, immunity, and endometrial microbiome exert an influence on the uterine mucosal barrier and embryo implantation, highlighting the need for further investigation into their intricate interrelationships.

### 3.4 Anatomy, histology, immunity, and microbiome of the fallopian tubes and their interactions

#### 3.4.1 Anatomy, histology, and immunity of the fallopian tubes

The fallopian tubes are a pair of slender and curved muscle tubes divided into four parts: the stroma, the isthmus, the ampulla, and the infundibulum. Their medial side is connected to the uterine horn, while their external end is free and umbrella-like (Eddy and Pauerstein, 1980). The fallopian tube is similar to other hollow organs, as its wall consists of sequential layers of mucosa, muscular, and serosa from the inside to the outside (Li and Winuthayanon, 2017). The mucosal epithelium of the fallopian tube comprises a single layer of tall columnar cells, which can be classified into three types: ciliated cells, secretory cells, and peg cells (Rigby et al., 2022). Among these types, ciliated cells and secretory cells are the main ones. Ciliated cells are primarily situated in the fimbriae of the uterine tube, accounting for more than 50% of its distribution, and secretory cells are mainly located in the isthmus, comprising approximately 60% of the entire mucosal epithelium, and are chiefly responsible for secreting active ingredients into the fallopian tube fluid (Pérez-Cerezales et al., 2018).

Immune cells are the prominent participants in the immune response of the fallopian tube and can be divided into innate immune cells and adaptive immune cells. These cells are mainly distributed in the epithelium and lamina propria of the fallopian tube, playing an indispensable role in establishing pregnancy and eliminating pathogens (Lee et al., 2015). The innate immune cells typically include NEUs, DCs, NK cells, MCs, and Mφs, but the dominant cell population has not been determined (Lee et al., 2015). According to most studies, T cells are described as the main population of immune cells in healthy fallopian tubes among the adaptive immune cells, accounting for 40%~60% of all leukocytes (Lee et al., 2015). In contrast, B cells constitute a relatively small proportion (5%~10%) (Lee et al., 2015). The epithelial cells forming the epithelial barrier of the fallopian tubes express cell receptors to detect pathogens and transmit defense response signals. Besides, they can also secrete cytokines and AMPs to stimulate and regulate immune responses (McGlade et al., 2022; Schleimer et al., 2007).

#### 3.4.2 Effects of the interactions between the microbiome and the anatomy, histology and immunity of the fallopian tubes on its physiological functions

The fallopian tube is the site where the ova and sperm combine and the channel for transporting the zygote (Li and Winuthayanon, 2017). The fluid environment secreted by the tubal secretory cells is rich in nutrients, such as proteins, adhesion molecules, specific glycoproteins, and inorganic salts, providing favorable conditions for microbial proliferation (Pérez-Cerezales et al., 2018). The mucosal epithelium of the fallopian tube has a highly developed ciliary structure, and its motility may be necessary for removing invasive microorganisms, guiding fertilization, and promoting zygote movement, which may be affected by pathogens (Barton et al., 2020). For instance, the fine cilia on ciliated cells are vulnerable to invasion and destruction by pathogens such as *Neisseria gonorrhoeae*, *Mycoplasma*, *Chlamydia*, and others, resulting in edema, necrosis, functional decline or loss of cilia (Barton et al., 2020). Yang et al. established a mice model of *Chlamydia* infection and observed the activation of complement factor 5 (C5) in the hydrosalpinx group, characterized by fallopian tube enlargement and fluid accumulation (Yang et al., 2014). Previous studies have suggested that C3 and C5 may initiate fibrotic responses in epithelial cells (Portilla and Xavier, 2021). Ciliary lesions and fibrosis cause inflammatory thickening of the fallopian tube wall and narrowing or even blockage of the lumen, which may lead to adverse reproductive outcomes such as tubal infertility and tubal pregnancy (Barton et al., 2020; Yang et al., 2014; Portilla and Xavier, 2021). Furthermore, animal experiments showed that *Lactobacillus rhamnosus* GG attenuated the pathological damage caused by *Chlamydial muridarium* infection in the fallopian tube (Zhou et al., 2021). This study suggests that *Lactobacillus* may help maintain the homeostasis of female fallopian tube tissue. However, the underlying interaction mechanisms between *Lactobacillus* and the histological and immune systems in the fallopian tube have been poorly studied.

## 4 Factors affecting the microbiome of the female reproductive tract

**Figure 3:**
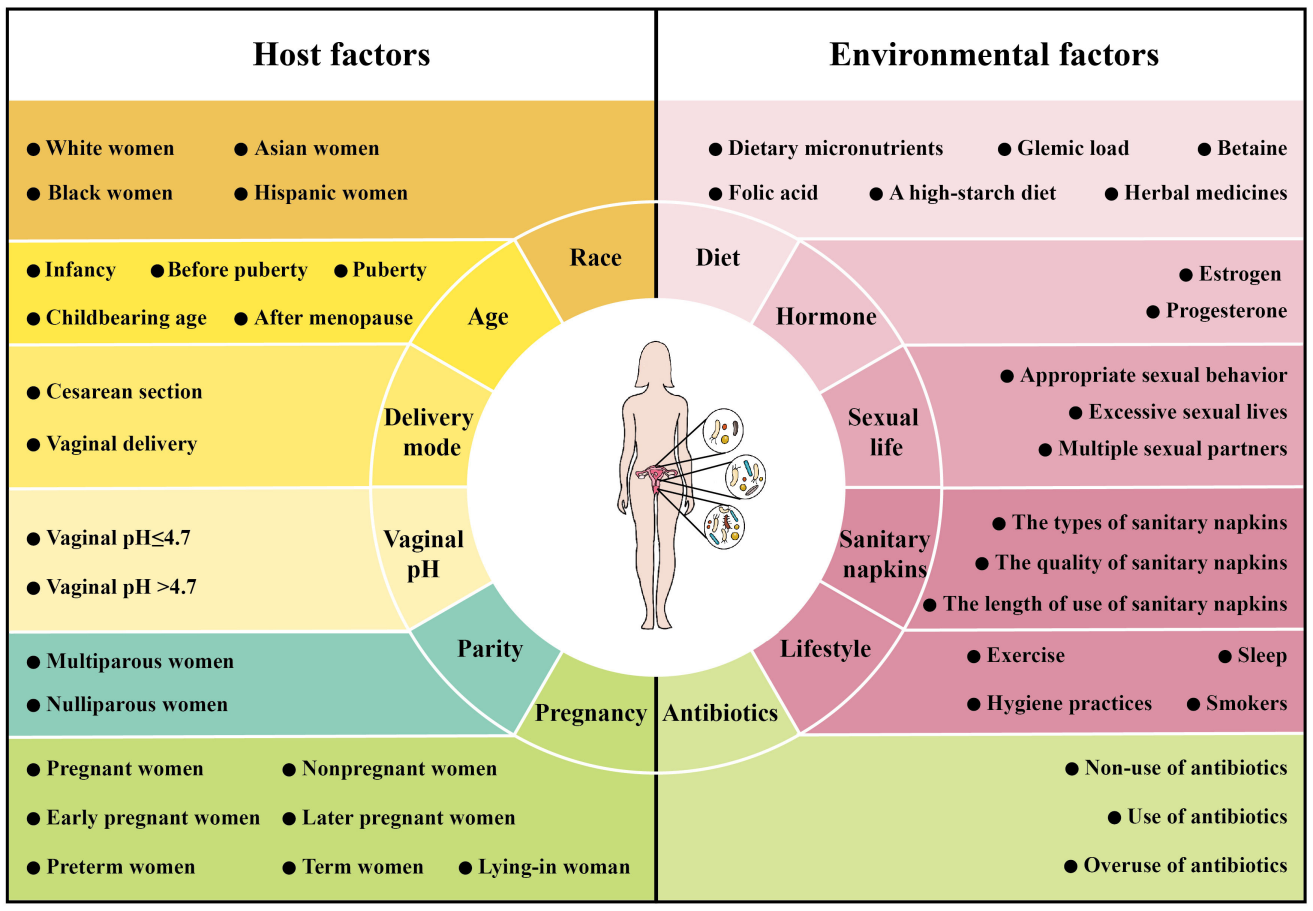
Factors affecting the microbiome of female reproductive tract. The composition of the female reproductive tract microbiome is influenced by host factors [race (including White women, Asian women, Black women and Hispanic women), age (including infancy, before puberty, puberty, childbearing age and after menopause), pregnancy (including pregnant women, nonpregnant women, early pregnant women, later pregnant women, preterm women, term women and lying-in woman), delivery mode (including cesarean section and vaginal delivery), parity (including multiparous women and nulliparous women) and vaginal pH (including vaginal pH ≤ 4.7 and vaginal pH >4.7)] and environmental factors [hormone (including estrogen and progesterone), diet (including dietary micronutrients, glycemic load, betaine, folic acid, a high-starch diet and herbal medicines), antibiotics (including non-use of antibiotics, use of antibiotics and overuse of antibiotics), sexual life (including appropriate sexual behavior, excessive sexual lives and multiple sexual partners), sanitary napkins (including the types of sanitary napkins, the quality of sanitary napkins and the length of use of sanitary napkins) and lifestyle (exercise, sleep, hygiene practices and smokers)].

The interaction between microbiome and host and environment can directly or indirectly alter the composition of the female reproductive tract microbiome (Noyes et al., 2018). Nevertheless, there is currently no consistent conclusion on the factors influencing the female reproductive tract microbiome (Łaniewski et al., 2020; Wang et al., 2022). In this section, we review the important factors that influence changes in the female reproductive tract microbiome, divided into host factors and environmental factors (**Figure 3**).

### 4.1 Host factors

#### 4.1.1 Race

The vaginal microbiome varies among women of different races. In terms of the vaginal microbial species, anaerobic bacteria tend to colonize the reproductive tract of White women, Asian women and Caucasian women, while *Candida* is more prevalent in colonizing the vaginal mucosa of Black women (Gupta et al., 2020). Similarly, the infection rates of *Mycoplasma*, *Ureaplasma urealyticum* and *Neisseria gonorrhoeae* were higher in Black women, and the separation rate of *Trachomatis* in the vagina in Black women and Hispanic women was significantly higher than that in White women (Newton et al., 2001). In terms of the vaginal microbial diversity, Black women and Hispanic women had significantly higher diversity than Asian women and White women (Ravel et al., 2011). In terms of the abundance of vaginal microbes, the vaginal microbiome dominated by *Lactobacillus* was found in White women (89.7%), Asian women (80.2%), Black women (61.9%) and Hispanic women (59.6%), and the abundance of *Lactobacillus* was only 37% in Black women in another study (Ravel et al., 2011; Anahtar et al., 2015). Other studies have found that BVAB in the vagina of Black women is 25.8% higher than that of White women, which may be linked to the increase in corticotropin-releasing hormone-related gene mutations (Newton et al., 2001; Ryckman et al., 2009). Currently, the specific reasons for the race-related differences in the vaginal microbiome are unknown. Generally, the differences may be related to host genetic characteristics, geographic environments, and lifestyles. Therefore, studying the effects of race on the microbiome requires further consideration of the mixed effects of additional variables, which can help to develop personalized microecological therapies.

#### 4.1.2 Age

The reproductive tract microbiome changes with the age of women and interacts with women throughout the lifespan. In infancy, the vaginal microbiome is dominated by a mixture of aerobic bacteria and anaerobic bacteria. Before puberty, anaerobic microbial communities dominate the vaginal microbiome. During puberty and childbearing age, the vaginal microbiome changes to be dominated by *Lactobacillus* (Ravel et al., 2011; Song et al., 2020). After menopause, the abundance of *Lactobacillus* in the vaginal microbiome gradually decreases, while that of *Escherichia coli* increases, leading to menopausal symptoms such as vaginitis and vaginal dryness (Marnach et al., 2022). Another study showed that uterine microbial diversity decreased with increasing age, while vaginal microbial diversity increased (Wang et al., 2021). The compositions of the vaginal microbiome and uterine microbiome underwent some changes with age, which may be related to estrogen levels. Overall, these findings suggest that age is a significant factor affecting the female reproductive tract microbiome and has an important impact on reproductive health. Exploring the relationship between age and the female reproductive tract microbiome could help alleviate age-related reproductive diseases.

#### 4.1.3 Pregnancy

Pregnancy is an important window for regulating the vaginal microbiome, and the effect of it has been proven. ① The diversity and richness of the vaginal microbiome in pregnant women are more stable in pregnant women than in nonpregnant women (Romero et al., 2014). ② In the early stage of pregnancy, *Lactobacillus* dominates the vaginal microbiome and persists throughout pregnancy. The abundance of *Lactobacillus* increases with gestational age, which may be related to changes in SCFA and hormone levels (Jost et al., 2014). ③ Compared with preterm women, term women have a richer vaginal microbial diversity with a higher abundance of *Lactobacillus* (except for *L. iners*) and a lower abundance of anaerobic bacteria (Aagaard et al., 2012; Lewis et al., 2017). ④ The vaginal microbiome undergoes significant alterations after normal delivery due to decreased estrogen levels and lochia discharge; this leads to a decrease in communities characterized by *Lactobacillus* and an increase in the diversity of anaerobic bacteria such as *Anaerococcus*, *Prevotella*, and *Peptoniphilus*, which may last up to one year postdelivery (DiGiulio et al., 2015; Severgnini et al., 2022). It is worth noting that changes in the vaginal microbiome during late pregnancy aid in increasing maternal energy storage, thus contributing to fetal growth and development (Wang et al., 2020). Accurately understanding the changes in the maternal vaginal microbiome during pregnancy can better ensure favorable maternal pregnancy outcomes and fetal health.

#### 4.1.4 Delivery mode

The mode of delivery (cesarean section or vaginal delivery) affects the gut microbiome of infants, and emerging research has also found its impact on the vaginal microbiome in adulthood (de Koff et al., 2022; Stennett et al., 2020). The relative abundance of *L. jensenii* and *L. iners* was higher in women who delivered vaginally, while the abundance of *Prevotella bivia* was higher in women who delivered by cesarean section (Stennett et al., 2020). Moreover, compared with women who delivered vaginally, women who delivered by cesarean section exhibited a threefold increase in the likelihood of having low-*Lactobacillus* CST IV. The elevation in this risk heightens susceptibility to sexually transmitted infections (STIs) and abnormal pregnancy outcomes (Stennett et al., 2020). The composition and relative abundance of *Lactobacillus* and other microbes in the vagina varied between women who underwent cesarean delivery and those who delivered vaginally, suggesting that vaginal delivery is an important method to enhance the reproductive tract health of the baby into adulthood.

#### 4.1.5 Parity

The composition of the vaginal microbiome and endometrial microbiome are closely correlated with parity. Kervinen et al. reported that parity was inversely associated with the abundance of *L. crispatus*. The relative abundance of *L. crispatus* exhibited a declining trend, with percentages of 58.1% in nulliparous women, 25.7% in women who had given birth once, and 15.4% in women who had given birth twice or more (Kervinen et al., 2022). Additionally, the relative abundances of *L. gasseri* and *L. iners* increased with higher parity (Kervinen et al., 2022). This study suggested that nulliparity was a significant contributing factor to the high abundance of *L. crispatus* associated with gynecological health. Similarly, Bogado et al. found that the abundences of *Fusobacterium* and *Bacillus* in the endometrial microbiome of cows were lower in multiparous cows than in nulliparous cows, while the abundences of *Bifidobacterium* and *Staphylococcus* were higher in multiparous cows (Bogado Pascottini et al., 2021). As a result, parity should be taken into account when studying the composition of the vaginal and endometrial microbiome and its effect on reproductive outcomes.

#### 4.1.6 Vaginal pH

The vaginal pH affects the composition of the vaginal microbiome, and in the population with a mean vaginal pH greater than 4.7, *Lactobacillus* is no longer the dominant microbiome (Ravel et al., 2011; Barrientos-Durán et al., 2020). Moreover, excessive changes in pH cause disorders of the vaginal microbiome, placing the body in a pathological state. For instance, vaginal irrigation can frequently disrupt the homeostasis of the microbiome, increase the vaginal pH, inhibit the growth of *Lactobacillus*, and lead to external bacterial invasion (Gajer et al., 2012). Vaginal irrigation can not only introduce exogenous substances that cause pH changes but can also promote mechanical cleaning of symbiotic bacteria, thus affecting the ecological balance of the vagina (Brotman et al., 2008). Notably, when studying the composition of the vaginal microbiome, it is necessary to consider the adaptive changes in the composition and types of female reproductive tract microbiome under different vaginal pH values.

### 4.2 Environmental factors

#### 4.2.1 Hormones

As hormone levels change during the menstrual cycle, the microbiome composition from the vagina to the uterus changes; compared to the secretory phase, the proliferative phase seems to be associated with bacterial proliferation in the vagina and endometrium, and the microbiome composition is less stable during this phase (Romero et al., 2014; Sola-Leyva et al., 2021). Shen et al. found that estrogen therapy in postmenopausal women with atrophic vaginitis significantly increased the relative abundance of *Lactobacillus* in the vagina (Shen et al., 2016). Additionally, the use of hormonal contraception (e.g., long-acting hormonal contraceptives, intrauterine devices) may have an adverse impact on the female reproductive tract microbiome, resulting in invasion and colonization by anaerobic microbes and *Actinomycetes*, and their long-term use can put women at greater risk of bacterial vaginosis (BV) and streptococcal infection (Vodstrcil et al., 2013; Whitney et al., 2021; Aubert et al., 1980). Taken together, the effects of hormones on the female reproductive tract microbiome should not be ignored and need to be further studied. Resolving this issue will contribute to regulating the female reproductive tract microbiome using hormones, reducing the occurrence of hormone-mediated female reproductive tract diseases, and promoting female reproductive health.

#### 4.2.2 Diet

Dietary habits are a complex psychosocial behavior that can easily be confused by socioeconomic factors. Despite this, it has long been considered that diet is related to the composition and function of the gut microbiota (Tilg, 2010). The gut microbiota glycolyses and ferments indigestible carbohydrates in the diet into short-chain fatty acids (SCFAs) (Amabebe and Anumba, 2020). Considering that the gut microbiota continues to migrate to the vagina and SCFAs may be transmitted to the vagina through the bloodstream, it is evident that diet has an impact on the vaginal microbiota (Amabebe and Anumba, 2020). Subsequent studies have shown that inadequate intake of dietary micronutrients, such as vitamins A, C, and E and β-carotene, as well as a high glycemic load in women of childbearing age are associated with an increased incidence of BV (Tohill et al., 2007). Conversely, the intake of betaine and folic acid can mitigate the risk of BV, and a high-starch diet may promote vaginal health by lowering the vaginal pH (Miller et al., 2016; Tuddenham et al., 2019). In summary, a good diet is vitally important for a healthy female reproductive tract microbiota, which contributes to maintaining reproductive health and improving reproductive capacity.

#### 4.2.3 Antibiotics

Antibiotics are inevitable in the fight against reproduction tract infections, and their use can suppresses the propagation of pathogenic bacteria and the development of diseases. In addition, the use of antibiotics during pregnancy can cause changes in the maternal reproductive tract microbiome, which have long-term effects on early neonatal microbial colonization (Romero et al., 2014). Overuse of antibiotics disrupts the normal reproductive tract microbiome, leading to the emergence of a large number of drug-resistant microorganisms (DRMs). Marnach et al. found that the overuse of antibiotics easily caused vaginal *Candida* infection, and the longer the use time was, the higher the probability of infection (Marnach et al., 2022). Overall, the rational use of antibiotics is a factor that must be considered to maintain reproductive health. Further research into how antibiotics interact with the reproductive tract microbiome will provide valuable insights into the use of antibiotics, which may help mitigate the negative effects of antibiotics.

#### 4.2.4 Sexual life

The transmission of beneficial microbiome occurs during sexual activity, which may confer advantages to both males and females (Noyes et al., 2018; Smith and Mueller, 2015). Not only is the dominance of *Gardnerella vaginalis* in women is significantly associated with reproductive tract inflammation in male partners, but other studies have confirmed that inappropriate sexual behavior can also seriously interfere with the balance of reproductive tract microbiome in women of childbearing age (Vodstrcil et al., 2017; Mändar et al., 2015). Kreisel et al. proposed that the incidence of sexually transmitted diseases is highest among women and man aged 15 to 24 in the United States, possibly because women and man in this age group may have unclean, active and excessive sexual lives (Kreisel et al., 2021). BV can be sexually transmitted from a women to a man and his female partner, and the incidence in women with more than 2 sexual partners was 1.77 times higher than that in women with only 1 sexual partner or no sexual partner (Chen et al., 2017; Faught and Reyes, 2019). Engaging in sexual activity with multiple sexual partners can destabilize the vaginal microbiome and reduce the relative abundance of *Lactobacillus*, thereby increasing the risk of BV transmission (Gajer et al., 2012; Jespers et al., 2015). Semen is an alkaline substance, and sexual intercourse will cause a temporary increase in the vaginal pH, which can be restored 8 hours after intercourse. If sexual intercourse occurs repeatedly within a day, the vaginal pH is always high, which is conducive to the growth of conditional pathogens (Tuddenham et al., 2021). Therefore, proper sexual habits are an important factor in maintaining reproductive tract health.

#### 4.2.5 Sanitary napkins

The composition of the vaginal microbiome may be influenced by the type of sanitary napkins used, and improper usage habits of sanitary napkins can lead to the colonization of pathogens in the reproductive tract. Hickey et al. compared the changes in the vaginal microbiome in mid-cycle menstrual and menstrual women by using two designated types of sanitary napkins. They found that neither type of sanitary napkins had a significant effect on the composition of the vaginal microbiome at different stages of the menstrual cycle (Hickey et al., 2013). Other studies have demonstrated that there were statistically significant differences in the prevalence of *Gardnerella vaginalis* and anaerobic gram-negative rods between the groups using the two different types of sanitary napkins during the premenstrual visit (Chase et al., 2007). Hence, it is necessary to control for the confounding factor of sanitary napkin brands when investigating the changes in the vaginal microbiome during menstruation. The use of sanitary napkin products during nonmenstrual periods may impede perineal air circulation, elevate local temperature and humidity, and potentially alter vaginal pH levels, thereby promoting the colonization and proliferation of *Candida* and *Staphylococcus aureus* (Marnach et al., 2022; Moosa et al., 2020). During the menstrual period, menstrual blood flows out of the vagina, and the congealed blood is a good culture medium for bacteria. At this time, if sanitary napkins with unqualified disinfection ratings are used or are used for too long, exogenous pathogenic bacteria are likely to reproduce in the menstrual blood and invade the reproductive tract (Zhang et al., 2021).

#### 4.2.6 Lifestyle

Exercise and sufficient sleep promote microbial homeostasis by reducing inflammatory responses, while poor modern lifestyles disrupt the balance of the reproductive tract microbiome. Drying clothes in poorly ventilated environments or wearing synthetic underwear, can lead to an imbalance in the microbial community, particularly *Candida* proliferation (Marnach et al., 2022). The rate of vaginal *Candida* colonization in women using vaginal irrigation fluid (26%) was higher than that in women without this habit (20%), and excessive vaginal irrigation may lead to vaginal microbiome imbalance, increasing the incidence of upper reproductive tract infections (Hyman et al., 2014; Barousse et al., 2004). Excessive cleaning of the vulvar area with potential irritants such as soaps, bubble baths, powders, or vaginal sprays can also affect the vaginal microbiome (Watson and Calabretto, 2007). In addition, smokers have a lower proportion of vaginal *Lactobacillus*, and smoking can increase the incidence of BV (Ding and Schloss, 2014; Brotman et al., 2014; Fettweis et al., 2014). Strengthening reproductive health education and developing a set of healthy lifestyle schemes for the entire population, are beneficial to maintaining the homeostasis of the reproductive tract microenvironment and improving health indicators.

## 5 Personalized medicine approaches in managing female reproductive tract health based on microbiome

Reproductive health is increasingly becoming a global concern. There is growing interest in the relationship between microbiome and reproductive health (Wang et al., 2022). Emerging evidence suggests that regulating the microbial composition of the reproductive tract to address dysbiosis represents a novel approach to managing female reproductive tract health (Łaniewski et al., 2020; Wang et al., 2022). This section focuses on microbiome-based approaches to personalized medicine, including antibiotics, microecological preparations, biofilm interference, vaginal microbiota transplantation.

### 5.1 Antibiotics

Antibiotics are extensively used to treat diseases of the female reproductive tract caused by pathogenic microorganisms, such as BV, cervicitis, endometritis, salpingitis, and others, thereby facilitating the restoration of homeostasis within the female reproductive tract (Tomás et al., 2020; Friedland et al., 1996; Kitaya et al., 2017; Walker et al., 1991). Metronidazole has been the first-line therapy for BV for a long time (Tomás et al., 2020; Bradshaw and Sobel, 2016). Metronidazole can reduce BVAB, including *Gardnerella*, *Atopobium*, and *Prevotella*, and cure approximately 60%~70% of women with BV within 4 weeks (Larsson and Forsum, 2005). However, the administration of metronidazole to BV patients also resulted in the establishment of a microbial community state dominated by *L.iners*, which is commonly associated with adverse reproductive outcomes, particularly an increasing likelihood of BV recurrence (Mtshali et al., 2021). A study demonstrated the significant contribution of the synergistic interaction between anaerobic bacteria and the formation of stubborn biofilms in the vagina to heightened drug resistance against metronidazole among BV patients (Tomás et al., 2020; Rosca et al., 2022). The high recurrence and drug resistance of metronidazole in the treatment of BV pose a formidable challenge, while tinidazole and clindamycin present promising alternatives to effectively address this issue (Li et al., 2020; Schwebke and Desmond, 2011). Some studies have shown that the tinidazole treatment group, at a lower dosage, cured 95%~97% of women with BV; the cure rate was higher than that of the metronidazole treatment group, while its recurrence rate and adverse reactions were significantly lower (Raja et al., 2016; Thulkar et al., 2012). The cure rates of clindamycin and metronidazole are comparable, but clindamycin exhibits relatively higher drug susceptibility and lower recurrence rates than metronidazole in the treatment of *Gardnerella vaginalis* (Li et al., 2020). While the early-stage recurrence rate of tinidazole and clindamycin is comparatively lower than that of metronidazole, there still exists a potential for recurrence within one year after treatment, with rates reaching up to 50% (Bradshaw and Sobel, 2016). Therefore, exploring more effective approaches to prolong or enhance the efficacy of antibiotics further and reduce the recurrence of BV is imperative.

Cervicitis can often be asymptomatic, and if left untreated, the incidence rate in average women is more than 25.5%, which can result in damage to the upper reproductive organs and lead to pelvic inflammatory diseases as well as infertility (Friedland et al., 1996; Salmanov et al., 2022a). The common pathogens responsible for cervicitis include *Neisseria gonorrhoeae*, *Chlamydia trachomatis*, and *Mycoplasma genitalium*, each requiring distinct treatment strategies (Marrazzo and Martin, 2007). The first-line drugs for treating gonococcal cervicitis are third-generation cephalosporins such as ceftriaxone or ceftriaxone, and azithromycin is recommended as a second-line therapeutic option (Friedland et al., 1996; Unemo et al., 2019). From 2009 to 2016, the prevalence of cefixime resistance for gonococcal cervicitis in Europe continued to decline, and the resistance to azithromycin showed a trend of first decreasing and then increasing (Stefanelli et al., 2017). In the drug sensitivity test, the minimum inhibitory concentration (MIC) of cephalosporins was 0.125mg/L, while the MIC of azithromycin was 1.0mg/L (George et al., 2019). In general, the drug resistance in gonococcal cervicitis is still not low, posing a significant global health threat. *Chlamydia trachomatis* is an important pathogen in non-gonococcal cervicitis (Marrazzo and Martin, 2007). Käding et al. showed that first-line antimicrobials, such as doxycycline (100mg twice daily for 7 days) and azithromycin (1g in a single dose), have been recommended for the treatment of *Chlamydia trachomatis* infection, with reported efficacy rates of 100% and 97% respectively (Käding et al., 2021). The commonly used therapeutic agents for *Mycoplasma genitalium* include macrolides (e.g., azithromycin, pristinamycin), tetracyclines (e.g., doxycycline, minocycline), and quinolones (e.g., moxifloxacin, sitafloxacin) (Jensen et al., 2021 2022). Currently, the prevalence of macrolide resistance in *Mycoplasma genitalium* exceeds 50% in numerous countries, while there is a significant upward trend in tetracycline resistance (Tagg et al., 2013; Salado-Rasmussen and Jensen, 2014). Durukan et al. demonstrated that the combination of doxycycline and azithromycin achieved a clinical cure rate of 95.4% for drug-resistant *Mycoplasma genitalium*. In comparison, the combination of doxycycline and moxifloxacin yielded a cure rate of 92.0% (Durukan et al., 2020). The study conducted by Read et al. obtained similar findings, and the statistical analysis of adverse reactions associated with the treatment above revealed that azithromycin exhibited the highest incidence of adverse reactions (91.4%), followed by doxycycline (86.6%) and sitafloxacin (80.5%) (Read et al., 2019). These studies suggest quinolones are more suitable for treating cervicitis caused by *Mycoplasma genitalium* infection.

Chronic endometritis (CE), characterized by persistent inflammation of the endometrium, has garnered significant attention due to its potential impact on reproductive outcomes (Chen P. et al., 2021). Doxycycline, a broad-spectrum antibiotic, has long been listed as the first-line therapy for CE worldwide (Kitaya et al., 2017). Kitaya et al. administered a 14-day course of doxycycline in RIF patients with CE, resulting in a cure rate of 92.3% for CE (Kitaya et al., 2017). Johnston-MacAnany et al. reported that the second-line therapy, which consisted of ciprofloxacin and metronidazole, also played an essential role in curing CE (Johnston-MacAnanny et al., 2010). Among patients pathologically diagnosed with CE but with negative endometrial pathogen culture, treatment with broad-spectrum antibiotics, including doxycycline, metronidazole, and ceftriaxone, resulted in persistent CE observed in 53.8% of patients at hysteroscopy and histology (Cicinelli et al., 2015). The study found that the clinical pregnancy rate of patients with persistent CE was 32% lower compared to those who experienced CE recovery, indicating that these broad-spectrum antibiotic treatments have certain limitations (Cicinelli et al., 2015). Furthermore, targeted antibiotic treatment for CE patients based on endometrial pathogen detection has been studied. For instance, amoxicillin combined with clavulanic acid was recommended for most cases with Gram-positive bacteria (*Enterococcus faecalis*, *Streptococcus agalactiae*, *Streptococcus bovis*, *Staphylococcus epidermidis*, *Staphylococcus aureus*, and *Streptococcus milleri*); ciprofloxacin was suggested for most cases with positive Gram-negative bacteria (*Escherichia coli*, *Candida*, and *Klebsiella pneumoniae*); josamycin and minocycline were effective in treating *mycoplasma*/*ureaplasma* infections (Cicinelli et al., 2015; Kuroda et al., 2021). Finally, 83.3% of CE patients with positive pathogen cultures were cured after targeted antibiotic treatment (Cicinelli et al., 2015). So, these findings suggested that targeted antibiotic treatment was more effective than broad-spectrum antibiotic therapy, thereby improving adverse pregnancy outcomes.

The current main drugs used for treating salpingitis include ceftriaxone, doxycycline, metronidazole, ofloxacin, moxifloxacin, azithromycin, and clindamycin (Ross et al., 2017 2018). Research has shown that the combination of multiple antibiotics typically improves the efficacy of antibiotic therapy by broadening the spectrum of pathogen coverage (Walker et al., 1991; The European Study Group, 1992). The clinical cure rate of the cephalosporin with doxycycline combination was 94%, effectively reducing *Chlamydia trachomatis*, *Neisseria gonorrhoeae*, *aerobic* and *anaerobic pathogens*, which had the advantages of favorable tolerability and minimal adverse effects (Walker et al., 1991). Additionally, other studies have found that the clindamycin with gentamicin combination satisfactorily eradicated *Chlamydia trachomatis* and *Neisseria gonorrhoeae*, exhibiting a comparable cure rate (87%) to the cephalosporin with doxycycline combination therapy group (84%) (The European Study Group, 1992).

### 5.2 Microecological preparations

Microecological preparations are a crucial supplementary method for managing reproductive tract health, and the most common type of microbial agent is probiotics, such as vaginal probiotic tablets, vaginal probiotic suppositories, and vaginal probiotic capsules (Recine et al., 2016; Cohen et al., 2020; Huang, 2017). A study discovered that the rate of symptom improvement in BV patients who used *Lactobacillus rhamnosus* BMX 54 vaginal tablets for 9 months was 92%, which was significantly higher than the rate observed in other patients (79%) (*P*<0.001) (Recine et al., 2016). Clinical trials have evaluated *L. crispatus* strain CTV-05 (administered as a vaginal suppository, known as LACTIN-V) for the treatment of BV (Cohen et al., 2020; Armstrong et al., 2022). In a randomized placebo-controlled phase 2b clinical trial involving women who were diagnosed with bacterial vaginosis and had completed vaginal metronidazole gel treatment, it was observed that the recurrence rate of bacterial vaginosis in the LACTIN-V group was significantly lower than that in the placebo group after 12 weeks (Cohen et al., 2020). These studies have confirmed that probiotics are essential for treating or preventing BV recurrence. In contrast, the synergistic combination of probiotics and antibiotics results in a more long-lasting treatment effect for BV (Recine et al., 2016; Cohen et al., 2020; Armstrong et al., 2022). In patients with cervical intraepithelial neoplasia (CIN) and high-risk HPV infection, the combination of placing vaginal *Lactobacillus* capsules, interferon α-2b, and loop electrosurgical excision procedure (LEEP) significantly increased the cure rate (90.48%) and the clearance rate of high-risk HPV (59.52%) compared to the control group that received only interferon α-2b and LEEP (73.81% and 40.48%, respectively) (Huang, 2017). The present study demonstrated that the administration of vaginal *Lactobacillus* capsules was associated with a higher rate of CIN cure and high-risk HPV clearance, along with a reduced amount of vaginal bleeding and postoperative complications (Huang, 2017). *Lactobacillus* also had cytotoxic effects on cervical cancer cell lines, inhibiting cancer cell proliferation and inducing its apoptosis (Kyrgiou et al., 2017; Wang et al., 2018). For example, Wang et al. cultured cervical cancer Caski cells using the supernatant of *Lactobacillus* and observed its inhibitory effect on the proliferation of Caski cells and induction of morphological changes (Wang et al., 2018). This study revealed that the supernatant of *L. crispatus*, *L. gasseri*, and *L. jensenii* could inhibit cervical cancer cell activity by suppressing the expression of HPV oncogenes and cell cycle-related genes (Wang et al., 2018). These findings suggest that probiotics have a potential contribution to the clearance of high-risk HPV and the treatment of cervical cancer. The cure rate of vaginal probiotic suppositories combined with antibiotics in RIF patients with endometrial microbiome dominated by non-*Lactobacillus* was 78.6%, which was significantly higher than that of antibiotics alone (33.33%) (Kadogami et al., 2020). Notablely, 131 RIF patients underwent microbial 16S rRNA gene sequencing before embryo transfer (study group), while 64 control group patients underwent embryo transfer without microbial analysis. Among them, 22.9% of patients in the study group detected abnormal endometrial microbiome and received personalized treatment with probiotics and antibiotics. The results showed that the cumulative pregnancy rate in the study group was higher than that in the control group (64.5% vs 33.3%, P<0.05) (Iwami et al., 2023). Therefore, the combination of vaginal probiotics and antibiotics may represent a promising therapeutic approach for treating RIF patients and improving their IVF outcomes, as supported by previous studies (Iwami et al., 2023; Kadogami et al., 2020). Overall, these findings demonstrate the feasibility of using vaginal probiotics to modulate the female reproductive tract microbiome and manage reproductive health.

### 5.3 Biofilm disruptors

Other promising novel therapies targeting BV or vaginal dysbiosis include TOL-463, a boric acid-based anti-infective with enhanced biofilm disruptive activity (Marrazzo et al., 2019). The results of a phase II clinical trial (n=106) demonstrated that treatment with TOL-463, in either vaginal gel or insert forms, is safe and well tolerated. Moreover, both the insert and gel formulations achieved significant clinical cure rates for BV, with 59% and 50%, respectively, observed on days 9-12 (Marrazzo et al., 2019). Current clinical evidence suggests that boric acid is a safe and alternative choice for the treatment of recurrent vulvovaginal candidiasis (Iavazzo et al., 2011). In the future, biofilm disruptors are expected to become a new strategy for treating BV and managing reproductive health.

### 5.4 Vaginal microbiota transplantation

Vaginal microbiota transplantation (VMT) refers to transplanting the vaginal microbiota from healthy women into the vaginas of BV patients, which can effectively restore the vaginal microbiota of patients and has achieved better results in regulating vaginal microbiota disorders. Chen et al. reported that VMT reduced the enrichment of IL-1β and TNF-α in vaginal tissues in animal experiments. At the same time, VMT increased the abundance of *Lactobacillus* while decreasing the numbers of *Enterobacter* and *Enterococcus*, thereby restoring the vaginal microbiota to normal levels and helping prevent the recurrence of vaginal dysbiosis (Chen T. et al., 2021). Other studies have further confirmed that VMT significantly improves the high drug resistance and susceptibility to recurrence of BV (Lev-Sagie et al., 2019; DeLong et al., 2019). A pilot study conducted in 2019 (n=5) demonstrated the feasibility of utilizing VMT from healthy donors as a therapeutic intervention for women suffering from intractable, antibiotic-unresponsive, and recurrent BV (Lev-Sagie et al., 2019). During the follow-up period of 5-21 months after VMT, four women treated with VMT achieved long-term remission, a significant improvement in symptoms and the reconstitution of a *Lactobacillus*-dominant microbiome (Lev-Sagie et al., 2019). Subsequently, three women received repeat VMT to achieve a lasting clinical response in the exploratory study (Lev-Sagie et al., 2019). No adverse effects associated with VMT were observed throughout the treatment, whereas the long-term consequences remain unknown (Lev-Sagie et al., 2019). The potential risks associated with this procedure, similar to other microbiome transplants, include the transfer of antimicrobial-resistant microorganisms and undetected pathogens (Bhutiani et al., 2018). Thus, it is imperative to establish stringent inclusion/exclusion criteria and conduct extensive testing of donor samples to minimize risks. The implementation of a screening approach for universal VMT donors has been described and successfully executed in another pilot study conducted in 2019, involving a sample size of 20 individuals (DeLong et al., 2019). Future studies involving larger cohorts and randomized, placebo-controlled designs will be necessary to determine the efficacy and durability of VMT.

## 6 Conclusion and outlook

Recent accumulated studies have shown that our understanding of the composition of the female reproductive tract microbiome in the female reproductive tract is still limited for several reasons: ① the collected samples may be susceptible to contamination; ② obtaining normal samples from the upper reproductive tract presents a challenge due to ethical restrictions and sampling difficulties; ③ microbial samples from the ovaries and fallopian tubes are mostly taken from patients with diseases in other parts of the reproductive tract; ④ researchers tend to overlook factors influencing the female reproductive tract microbiome. These factors make it difficult and challenging to accurately reveal the composition of the female reproductive tract microbiome. We summarized the current research on the female reproductive tract microbiome and preliminarily identified variations in its composition under different states. The specific findings are shown in **Table 2**.

**Table 2:** The composition of female reproductive tract microbiome in different states.

Anatomical region	Sample Type	Harbored Bacteria			References
		Healthy	Inflammatory	Cancerous	
Vagina	Vaginal swab	*L. crispatus*, *L. gasseri*, *L. iners*, *L. jensenii*, *Prevotella*, *Sneathia*, *Staphylococcus*, *Veillonella*, *Streptococcus*	*L. crispatus*↓, *L. jensenii*↓, *L. gasseri*↓, *L. iners*, *Prevotella*, *Gardnerella*, *Atopobium*, *Sneathia*, *Megasphaera*, *Mageeibacillus*, *Eggerthella*, *Leptotrichia*, *Shuttleworthia*, BVAB 2, *Parvimonas*	No description	(Chen et al., 2017; Muzny et al., 2020; Ravel et al., 2011; Santella et al., 2022; Łaniewski et al., 2018; Shipitsyna et al., 2013; Tamrakar et al., 2007; Zinsli et al., 2023)
Cervix	Cervical swab	*Lactobacillus* (*L. iners-*dominated), *Prevotella*, *Gardnerella*, *Sneathia*, *Streptococcus*, *Shewanella*, *Arthrobacter*, *Sphingobium*, *Sphingomonas*	*L. jensenii*↓, *Trichomonas*, *Mycoplasma*, BVAB3, *Neisseria*, *Chlamydia*	*Lactobacillus*↓, *Porphyromonas*, *Prevotella*, *Campylobacter*, *Sneathia*, *Fusobacterium*	(Chen et al., 2017; Onywera et al., 2019a; Onywera et al., 2019b; Łaniewski et al., 2018; Audirac-Chalifour et al., 2016; Carneiro et al., 2020; Gorgos et al., 2015; Lusk et al., 2016; Roy et al., 2021; Shroff, 2023; Wu et al., 2021)
Endometrium	Endometrium; Endometrial fluid	*Lactobacillus*, *Pseudomonas*, *Acinetobacter*, *Vagococcus*, *Sphingobium*, *Arthrobacter*, *Dysgonomonas*, *Shewanella*, *Pseudomonadaceae*, *Delftia*, *Sphingomonas*, *Erysipelothrix*	*Proteobacteria*↓, *Gardnerella*, *Streptococcus*, *Neisseria*, *Dialister*, *Bififidobacterium*, *Staphylococcus*, *Enterococcus*, *Prevotella*, *Sphingomonas*, *Enterobacteriaceae*, *Klebsiella*, *Phyllobacterium*, *Anaerococcus*, *Actinobacteria*, *Acinetobacter*	*Lactobacillus*↓, *Prevotella*, *Klebsiella*, *Atopobium*, *Dialister*, *Muribaculum*, *Pelomonas*, *Nocardioides*, *Anaerostipes*, *ph2*, *Treponema*, *Bacteroides*, *Arthrospira*, *Peptoniphilus*, *1-68*, *Ruminococcus*, *Porphyromonas*, *Anaerotruncus*, *Bacteroides*, *Pseudomonas*	(Moreno et al., 2016; Chen P. et al., 2021; Franasiak et al., 2016; Verstraelen et al., 2016; Walther-António et al., 2016; Chen W. et al., 2021; Kimura et al., 2019; Kitaya et al., 2018; Li et al., 2021; Liu et al., 2019; Medina-Bastidas et al., 2022; Moreno et al., 2018)
Fallopian tube	Fallopian tube tissue	*Shigella*, *Bacteroides*, *Staphylococcus*, *Enterococcus*, *Corynebacterium*, *Lactobacillus*, *Pseudomonas*, *Erysipelothrix*, *Facklamia*	*Chlamydia*, *Neisseria*, *Mycoplasma*, *Anaerobes*, *Staphylococcus*, *Escherichia*, *Klebsiella*, *Streptococcus*	*Chlamydia*	(Chen et al., 2017; Walther-António et al., 2016; Pelzer et al., 2018a; Kinghorn et al., 1986; Laban et al., 2019; Reekie et al., 2019; Salmanov et al., 2022b)
Ovary	Ovarian tissue; Follicular fluid	*Lactobacillus*, *Streptococcus*, *Staphylococcus*, *Enterococcus*, *Candida*, *Actinomyces*, *Fusobacterium*, *Peptostreptococcus*, *Propionibacterium*, *Oligotrophomonas*, *Xanthomonas*	*Escherichia*, *Enterobacter*, *Klebsiella*, *Staphylococcus*, *Pseudomonas*, *Enterococcus*, *Proteus*, *Streptococcus*, *Acinetibacter*, *Chlamydia*, *Mycoplasma*	*Lactobacillus*↓, *Acinetobacter*, *Chlamydia*, *Mycoplasma*, *Staphylococcus*, *Sphingomonas*, *Enterococcus*, *Chryseobacterium*, *Burkholderia*, *Francisella*, *Treponema*, *Corynebacterium*, *Blautia*, *Escherichia*, *Trabulsiella*	(Miles et al., 2017; Zhou et al., 2019; Walther-António et al., 2016; Banerjee et al., 2017; Salmanov et al., 2022b; Xie et al., 2017)

BVAB, BV-associated bacteria; ↓, decrease.

The microbiome interacts complexly with the anatomy, histology, and immunity of the female reproductive tract. Here, we focus on the effects of their interactions on the physiological functions of the reproductive tract. In general, the normal tissue structure of the vagina, cervix, uterus, and fallopian tubes provides a nutrient-rich habitat for the colonization and proliferation of tissue-resident microbiome, and their mucosa also regulates changes in the composition of the microbiome. The tissue-resident microbiome can secrete relevant metabolites to protect the mucosal epithelial barrier, thereby reducing the risk of reproductive tract infections. Pathogenic bacteria in the cervix may alter reproductive tract tissue structure and endanger human pregnancy through premature cervical remodeling. Pathogenic bacteria and their associated inflammation can disrupt the balance between Th17/Tregs in the endometrium and alter the expression levels of related cytokines, resulting in RIF of the embryo. Additionally, pathogenic bacteria can cause epithelial cell necrosis and death, as well as tubal cilia edema, necrosis, functional decline or loss through oxidative stress and inflammatory pathways. The reproductive tract mucosa senses and contacts pathogenic bacteria, thus activating the signaling cascade of immune cells, which is conducive to promoting the recruitment, growth and differentiation of mucosal immune cells to clear out pathogenic bacteria, maintaining the dynamic balance of reproductive tract microecology. The interaction between the microbiome and the anatomy, histology and immunity of the reproductive tract affects its physiological functions, including fertilization, embryo implantation, fetal development, fetal delivery and defense against pathogen infection.

The composition of the female reproductive tract microbiome is susceptible to host and environmental factors, and exposure to these factors can lead to dynamic physiological changes in the reproductive tract microbiome. Currently, most studies have focused on cross-sectional analyses of the correlations among the reproductive tract microbiome, host, and environment, and there is a lack of relevant longitudinal studies and data. In the future, prospective studies are urgently needed to analyze the influencing factors of the reproductive tract microbiome, especially the changes in the female reproductive tract microbiome in different populations and physiological stages, to more truly reflect the reproductive tract health status of women of childbearing age. The worsening host and environmental factors of the female reproductive tract microbiome, such as excessive changes in pH value, frequent intercourse, multiple sexual partners, and vaginal irrigation, may lead to the disturbance of the microbiome. The long-term disturbance of the microbiome in the reproductive tract is closely associated with the occurrence and development of female reproductive tract diseases, such as infections and malignant tumors. It is necessary to intervene in the case of adverse environmental factors to prevent the occurrence of female reproductive tract diseases.

Emerging evidence suggests that the compositions of the cervical, vaginal and endometrial microbiome can serve as a predictive and screening tool for various female reproductive tract diseases and pregnancy outcomes. The cervical and vaginal microbiome exhibit potential as predictive biomarkers for preterm birth, as well as screening and diagnostic tools for cervical HPV infection. Similarly, the endometrial microbiome also serves as a reliable biomarker for predicting reproductive success and screening for endometrial diseases. Evidence has shown that personalized medicine approaches based on microbiome provides the possibility for the precision treatment of some female reproductive tract diseases, and its standardized treatment has received increasing attention from experts and scholars in managing female reproductive tract health fields. Finally, further exploration should be conducted on the mechanisms of the microbiome and their metabolites in the physiological functions of the reproductive tract, as well as the prevention, monitoring and treatment methods of female reproductive tract diseases based on the microbiome.

## Author contributions

HG: Writing – original draft, Writing – review & editing. QL: Writing – original draft. XW: Writing – original draft. TL: Writing – original draft. HL: Writing – original draft. GL: Writing – original draft. LT: Writing – original draft. YC: Writing – original draft.
